# *Phocaeicola dorei* ameliorates progression of steatotic liver disease by regulating bile acid, lipid, inflammation and proliferation

**DOI:** 10.1080/19490976.2025.2539448

**Published:** 2025-08-03

**Authors:** Jieun Choi, Ye Rin Choi, Min Kyo Jeong, Hyun Ho Song, Jeong Seok Yu, Seol Hui Song, Jeong Ha Park, Min Ju Kim, Hyunjoon Park, Young Lim Ham, Sang Hak Han, Dong Joon Kim, Do Yup Lee, Ki Tae Suk

**Affiliations:** a Department of Agricultural Biotechnology, Seoul National University, Seoul, Republic of Korea; b Institute for Liver and Digestive Diseases, Hallym University, Chuncheon, Republic of Korea; c Department of Nursing, Daewon University College, Jecheon, Republic of Korea; d Department of Pathology, Hallym University College of Medicine, Chuncheon, Republic of Korea; e Green Bio Science & Technology, Bio-Food Industrialization, Seoul National University, Pyeongchang-gun, Republic of Korea

**Keywords:** Metabolic dysfunction-associated steatotic liver disease, gut, microbiota, metabolites, *Phocaeicola dorei*

## Abstract

Gut microbiota and their metabolites are known to influence the pathogenesis and progression of metabolic dysfunction-associated steatotic liver disease (MASLD). In this study, we investigated the potential beneficial effects of *Phocaeicola dorei* in modulating MASLD progression, beginning with clinical observations and followed by mechanistic validation in animal models. Human data (49 healthy controls and 129 MASLD patients) were collected to investigate gut microbial biomarkers. The relative abundance of *P. dorei* was found to significantly vary with MASLD severity in human. Western diet-induced MASLD mice supplemented *with P. dorei* (12 weeks, 10^9^ CFU/g twice/week) or 100 μl of *P. dorei* cell-free supernatant (CFS, 5 times/week) were utilized. STAM^TM^ mice (10 weeks, 10^8^ CFU/g four times/week) and RAW 264.7 cells were used for the validation. MASLD severity was determined based on liver/body weight, pathology, and biochemistry markers. Cecum feces were collected for 16S rRNA gene sequencing and metabolite profiles. In the animal model, *P. dorei* oral administration and its CFS alleviated lipid accumulation by increasing β-oxidation gene expression and inhibited inflammatory response from fatty liver to hepatitis progression. In the STAM^TM^ model, *P. dorei* decreased nuclear atypia and cell proliferation. Additionally, *P. dorei* CFS inhibited TNF-α and CXCL10 in activated macrophages, and this result was consistent with the results of animal models. *P. dorei* and its metabolites ameliorate MASLD progression by modulating bile acid, lipid accumulation, inflammation, and proliferation. *P. dorei* could be a promising candidate for novel microbiota-based therapeutic strategies against MASLD.

## Graphical abstract

**Figure:**
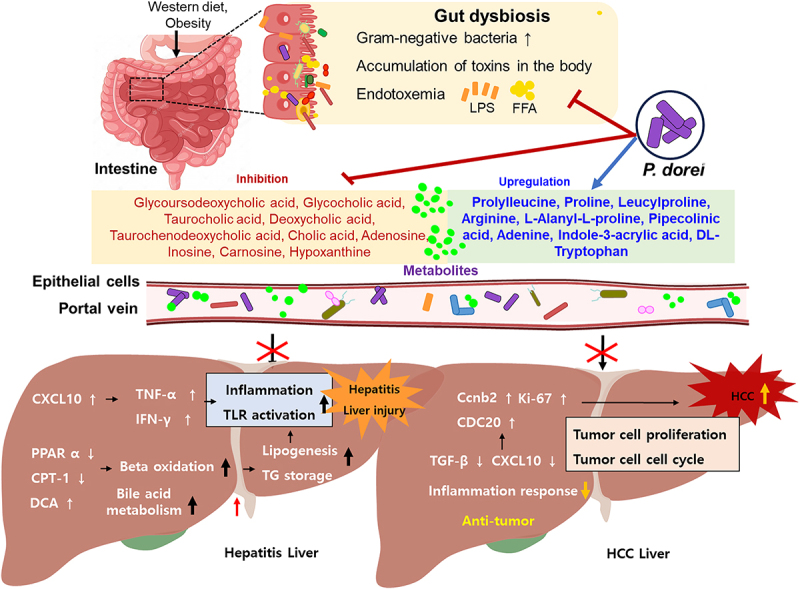

## Background

Metabolic dysfunction-associated steatotic liver disease (MASLD) is the most common chronic liver disease worldwide.^1^ It encompasses a spectrum ranging from hepatic steatosis to metabolic dysfunction-associated steatohepatitis (MASH), ultimately leading to liver cirrhosis and cancer.^2,3^ Approximately 10%-25% of people suffering from MASLD can progress to MASH, and it is estimated that 10–15% of patients with MASH will progress to hepatocellular carcinoma (HCC).^4^ MASLD is associated with metabolic syndrome, obesity, type 2 diabetes, and dyslipidemia; the global prevalence is estimated to be 24–30% in epidemiological studies. Approximately 90% of patients with MASLD accompany at least one feature of metabolic syndrome.^5^

The gut microbiome is essential for properly functioning and regulating the immune system, detoxification, and digestion of the host.^6–8^ Several studies have provided evidence that the gut microbiota is correlated with host metabolism and may play an important role in MASLD.^9,10^ Gut dysbiosis contributes to obesity-related disorders, pro-inflammatory activity, and immune imbalances, which are implicated in MASLD pathogenesis.^11,12^ Therefore, discovering and understanding the specific role of the intestinal microbiota in MASLD is required.

*Phocaeicola* (*Bacteroides*) *dorei* is found primarily in the intestinal tract of humans and animals.^13^ It has been reported that oral administration of *P. dorei* reduces gut lipopolysaccharide (LPS) levels and attenuates atherosclerosis in mice.^14^ Given that *Phocaeicola* treatment strengthens the intestinal barrier and modulates inflammation-related pathways, it may ameliorate the onset and progression of obesity-related metabolic diseases and MASLD. Recent studies have demonstrated that *P. dorei* exhibits strong anti-inflammatory activity, likely due to its structurally unique lipopolysaccharides, and contributes to improved cholesterol metabolism and reduced gut permeability.^15^ Additionally, *P. dorei* has been shown to interact with other gut microbes to enhance the production of protective metabolites such as short-chain fatty acids (SCFAs) and strengthen epithelial barrier function.^16,17^

We observed a significant increase in *P. dorei* abundance at the species level following in the feces of patients with fatty liver and hepatitis compared with healthy individuals. Considering these points, it can be hypothesized that *P. dorei* can play key-roles in the progression of the MASLD. The aims of this study are to determine whether *P. dorei* is related to the prevention effect in the progression of MASLD and to explore whether it is a promising pharmabiotics candidate for microbial therapeutic approaches to MASLD.

## Materials and methods

### Patients and healthy controls

A prospective cohort study was conducted from April 2017 to March 2020, registered under ClinicalTrials.gov (NCT04339725). The diagnosis of MASLD was established following criteria outlined in steatotic liver disease nomenclature guidelines.^18,19^ MASLD was defined as the presence of hepatic steatosis in conjunction with one cardiometabolic risk factor and no other discernible cause, comprises the most common causes of steatotic liver disease. Persons with MASLD and steatohepatitis will be designated as MASH. Diagnostic criteria included cardiometabolic factors such as body mass index, fasting glucose levels, medication history, blood pressure, and cholesterol levels. Individuals in the control group were those without any cardiometabolic abnormalities.

The MASH group comprised patients exhibiting elevated liver enzymes [aspartate aminotransaminase (AST) or alanine aminotransaminase (ALT) ≥50 IU/L] or histological evidence of hepatitis, without a history of excessive alcohol consumption (defined as > 210 g/week for males and > 140 g/week for females). Patients with autoimmune liver conditions, alcohol use disorder, pancreatitis, hemochromatosis, viral hepatitis, pregnancy, Wilson’s disease, drug-induced liver injury, or other malignancies were excluded from the study. Cirrhosis diagnosis was confirmed through the presence of related complications (e.g., varices, ascites, encephalopathy), blood test results, imaging findings, fibroscan assessments, or liver biopsy results.^20^ Furthermore, individuals using medications (antibiotics, proton pump inhibitors, probiotics, prebiotics, or chemotherapeutics) known to influence gut microbiota were excluded from the study at the time of enrollment. The control group consisted of healthy participants who visited the medical facility for routine health checkups. This research adhered to the ethical principles outlined in the 1975 Declaration of Helsinki and received approval from the institutional review board of all participating hospitals (approval number: 2016–134). Written informed consent was obtained from all study participants prior to inclusion.

### Interventional animal study

Six-week-old male C57Bl/6J mice were obtained for the MASLD animal experiment from Dooyeol Biotech (Seoul, Republic of Korea). The animals received human care, and all procedures were performed in accordance with the National Institutes of Health Guidelines for the Care and Use of Laboratory Animals. According to university guidelines, the mice were housed in a conventional animal facility at a temperature range of 21–24°C, dark/light cycle of 12 h, and humidity range of 40–50%. Mice had free access to water and food throughout the experiment and were monitored daily. After one week of acclimatization on a normal chow diet, the mice were fed a Western diet. For oral administration, mice were gavaged twice a week with 10^9^ CFU/100 ul *P. dorei* or *L. lactis*. We purchased the Western diet from Dooyeol Biotech (TD88137, Seoul, Korea), and the proportions of the diet were 42% fat, 42.7% carbohydrate, and 15% protein. All procedures were approved by the Institutional Animal Care and Use Committee of the College of Medicine, Hallym University (2019–30).

### STAM mouse model

Pathogen-free 14-day pregnant C57BL/6J mice were purchased from Dooyeol Biotech (Seoul, Korea), and 2-day-old male pups were injected 200 µg of streptozotocin (Sigma, MO, USA) for reduce insulin secretory capacity and fed a Western diet from the age of 4 weeks.^21^ This mouse model progresses MASH at 8 weeks of age and develops HCC at 16 weeks of age. Probiotics were suspended in PBS, and oral gavage feeding started at 5 weeks of age with a concentration of 10^9^ CFU/mouse three times per week. All procedures were approved by the Institutional Animal Care and Use Committee of the College of Medicine, Hallym University (2020–39).

### Cecal metabolites profiling of LC-Orbitrap MS

The dried extracts were reconstituted with 50 µl of 70% acetonitrile for LC-Orbitrap MS analysis. Chromatographic separation was performed using an Ultmate-3000 UPLC system (Thermo Fisher Scientific, Waltham, MA, USA) coupled with a 150 × 2.1 mm UPLC BEH 1.7 μm C18 column (Waters, Milford, MA, USA) and a 5.0 mm × 2.1 mm UPLC BEH 1.7 μm C18 VanGuard Pre-Column (Waters, Milford, MA, USA). The mobile phase consisted of buffer A (0.1% formic acid in water) and buffer B (0.1% formic acid in 100% acetonitrile). The flow rate was maintained at 0.35 ml/min with the following gradient profile: equilibration at 3% buffer B for 1 minute, a linear gradient from 3% to 100% buffer B over 9 minutes, 100% buffer B held for 1 minute, followed by re-equilibration at 3% buffer B for 3 min.

Mass spectrometry analysis was carried out using a Q-Exactive Plus Orbitrap instrument (Thermo Fisher Scientific, Waltham, MA, USA) operating in polarity-switching mode. Full MS scans were acquired within a mass range of 50–750 m/z at a resolution of 70,000 FWHM at m/z = 200, with an automatic gain control (AGC) target of 1e6 ions and a maximum injection time (IT) of 100 ms. Data-dependent MS/MS was performed on pooled samples for each ionization mode. The MS/MS settings were as follows: Top 5 MS1 ions; resolution 17,500 at 200 m/z; AGC target, 1e5; maximum IT, 50 ms; isolation window, 1.0 m/z; normalized collision energy (NCE), 30; intensity threshold, 2e3 ions; apex trigger, 3–6 s; dynamic exclusion, 5 s. Inclusion lists were prepared with m/z values and retention times corresponding to bile acids, indoles, and trimethylamine-related compounds.

### Cultured media metabolites profiling of LC-Orbitrap MS

Dried extracts were reconstituted with 50 µl of 80% methanol for LC-Orbitrap MS analysis. Chromatographic separation was performed using a Vanquish UPLC system (Thermo Fisher Scientific, Waltham, MA, USA) coupled with a 150 × 2.1 mm UPLC BEH 1.7 μm C18 column (Waters, Milford, MA, USA) and a 5.0 mm × 2.1 mm UPLC BEH 1.7 μm C18 VanGuard Pre-Column (Waters, Milford, MA, USA). The mobile phase was composed of buffer A (0.1% formic acid in water) and buffer B (0.1% formic acid in 100% acetonitrile). A flow rate of 0.35 ml/min was applied with the following gradient: equilibration at 10% buffer B for 2 min, increasing from 10% to 95% buffer B over 18 min, maintaining 95% buffer B for 5 min, and re-equilibrating at 10% buffer B for 5 min.

Mass spectrometric analysis was conducted using a Q-Exactive Focus Orbitrap (Thermo Fisher Scientific, Waltham, MA, USA) in polarity-switching mode. Full MS scans were performed over a mass range of 50–750 m/z with a resolution of 70,000 FWHM at m/z = 200, AGC target of 1e6 ions, and a maximum IT of 100 ms. Data-dependent MS/MS analysis was performed on pooled samples under each ionization mode. The parameters were: Top 3 MS1 ions; resolution 17,500 at 200 m/z; AGC target, 5e4; maximum IT, 50 ms; isolation window, 2.0 m/z; NCE, 30; intensity threshold, 1e5 ions; apex trigger, 3–7 seconds; dynamic exclusion, 5 seconds.

### Data processing and bioinformatics

Data acquisition and preprocessing were carried out using Xcalibur software (Thermo Fisher Scientific, San José, CA, USA). The acquired RAW files were processed with Compound Discoverer software (Thermo Fisher Scientific, San José, CA, USA) using a workflow comprising Spectra Selection, Retention Time Alignment, Unknown Compound Detection, Compound Grouping, Gap Filling, and mzCloud Search. The mass tolerance for MS1 at each step was set to 5 ppm. The Retention Time Alignment node was configured with a maximum shift of 1 minute. Compound identification was performed using mzCloud, with MS2 mass tolerance set to 10 ppm and an assignment threshold of 70%.

SIMCA 17 (Umetrics AB, Umea, Sweden) was utilized for multivariate statistical analyses, including partial least squares discriminant analysis. Bar graphs and Volcano plots were generated using GraphPad Prism software version 8.0 (GraphPad Software Inc., San Diego, CA, USA). Metabolic profiling patterns were evaluated using Spearman rank correlation via the corr.test function in the psych package in R. The final cluster number was determined to be five using the cascadeKM function from the vegan package, applying K-means partitioning across a range of K values. Visualization of metabolite patterns was performed using the ggplot2 package. Additionally, the UpSet function from the ComplexHeatmap package in R was employed to conduct regression analysis on key metabolites, with abundance patterns visualized using ggplot2.

Statistical analyses were performed for all continuous variables obtained from LC-MS data. Differences between groups were assessed using Student’s T-test. Hierarchical clustering analysis was conducted using Spearman rank correlation in MetaboAnalyst 6.0. A treemap was generated in Microsoft Excel (Microsoft, Seattle, WA, USA) based on compound classification from the Human Metabolome Database (HMDB).^22^ The metabolic network map was constructed using structural similarity (Tanimoto score) and biochemical linkage (KEGG reaction pair data) and visualized with a prefuse force-directed layout in Cytoscape version 3.9.1.^23^ SIMCA 17 (Umetrics AB, Umea, Sweden) was utilized for multivariate statistical analyses, including partial least squares discriminant analysis. Bar graphs and Volcano plots were generated using GraphPad Prism software version 8.0 (GraphPad Software Inc., San Diego, CA, USA). Metabolic profiling patterns were evaluated using Spearman rank correlation via the corr.test function in the psych package in R. The final cluster number was determined to be five using the cascadeKM function from the vegan package, applying K-means partitioning across a range of K values. Visualization of metabolite patterns was performed using the ggplot2 package. Additionally, the UpSet function from the ComplexHeatmap package in R was employed to conduct regression analysis on key metabolites, with abundance patterns visualized using ggplot2.^24^

## Results

### Cohort description

**Figure 1.**
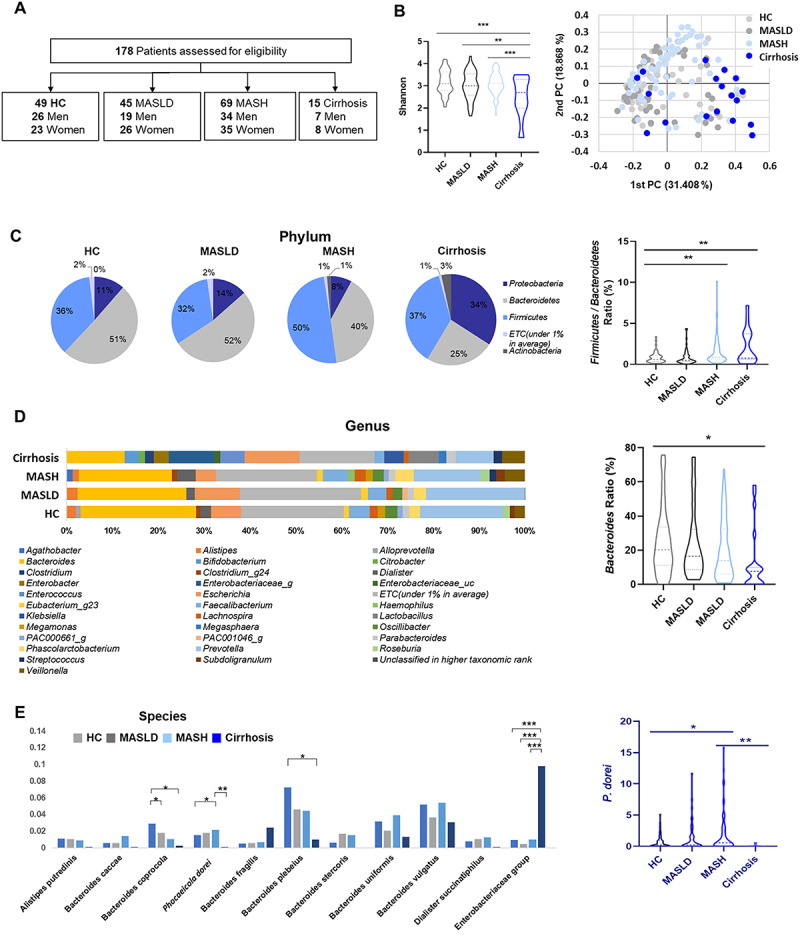
Taxonomic profile of gut microbiota according to the progression of MASLD. (A) Study design. (B) Box plots represent alpha diversity based on the Shannon index in different clinical conditions. Beta diversity was generated using relative OUT abundance data according to the UniFrac. (C) The pie chart shows relative abundance at the phylum level within each group. (D) Genus-level difference among groups. (E) RAs of *P. dorei* according to disease progression. All data are expressed as the means with SEM.

A total of 178 subjects were included in the study cohort and the characteristics of patients are documented (Table S1 and Figure 1A). MASH patients had the highest levels of body mass index (BMI), ALT, creatine, cholesterol, and triglycerides (TG) compared to the other groups. Cholesterol and TG levels increased with progression to MASLD and MASH compared to the normal control group, and both decreased in cirrhosis. The levels of AST and gamma-glutamyl transferase (GGT) were highest in cirrhosis patients, and the levels of ALT, creatine, cholesterol, and TG were decreased compared to MASH patients. High-density lipoprotein (HDL) tended to decrease in the order of MASH and cirrhosis.

### The proportion of P. dorei is correlated with the progression of MASLD

The gut microbiota of patients was profiled via 16S rRNA amplicon sequencing and taxonomic classification. Alpha diversity from the Shannon index showed significant decreases in MASLD, MASH, and cirrhosis order. In the beta diversity analysis, the dispersion of the cirrhosis group tended to be relatively less and was opposite to that of the HC group (Figure 1B). At the phylum level, the composition of the *Bacteroidetes* was reduced in MASH (40%) and cirrhosis (25%) compared to the HC (51%) (Figure 1C), *Proteobacteria* was significantly increased in cirrhosis (34%) (Figure 1D). We discovered that *P. dorei* was markedly increased in MASH patients compared to HC (*p* = 0.024), and extremely decreased in cirrhosis patients compared to MASH (*p* = 0.0089) (Figure 1E).

### Oral administration of P. dorei on the Western diet-induced MASH mice model

**Figure 2.**
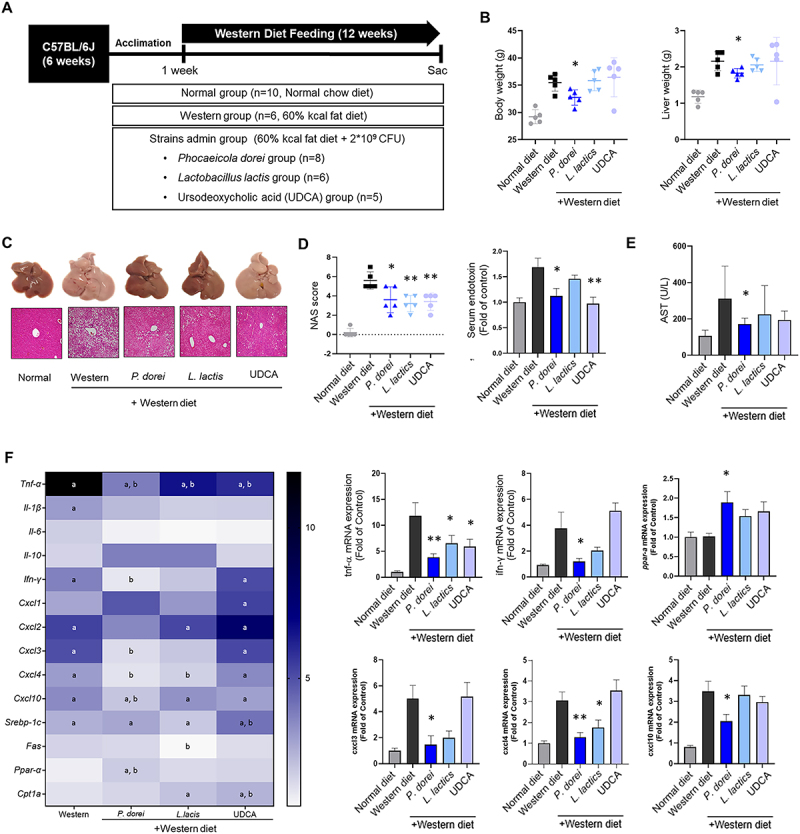
Oral administration of *P. dorei* and probiotics improves the Western diet induced liver damage. (A) Scheme of the animal experiment. (B) Body weight and liver weight. (C) Representative images of liver tissues and H&E staining (D) NAFLD activity scores on liver tissues (*P. dorei*, *p* = 0.0111; *L. lactis*, *p* = 0.0023; UDCA, *p* = 0.0050). Serum endotoxin biochemistry analysis. (E) Serum liver enzyme analysis (F) Gene expression of hepatic proinflammatory cytokines and lipid metabolism in mice liver. Data are expressed as the means with SEM. * *p* < 0.05, ** *p* < 0.01.

*P. dorei* ameliorated Western diet-induced mouse fatty liver and downregulated mRNA expression of hepatic inflammation by promoting lipid oxidation (Figure 2). Mice were acclimated for 1 week on a normal chow diet, then administered *P. dorei*, *Lactobacillus lactis*, and ursodeoxycholic acid (UDCA) to C57BL/6 mice 2 times per week 100 µl (2 X 10^9^CFU/mouse in PBS) with Western diet for 12 weeks (Figure 2A). UDCA was used as a positive control in the experimental model due to its hepatoprotective effects. The effect of the Western diet in inducing fatty liver was clearly demonstrated by H&E staining. Body weight, liver weight, and blood biochemical measurements did not show statistically significant differences in the tested groups (Figure 2B,C). In the NAS, the Western diet group was significantly increased (5.6 ± 0.8) compared with the normal diet group (0.2 ± 0.4) (*p* < 0.001) (Figure 2D). Conversely, *P. dorei* (3.6 ± 1.2), *L. lactis* (3.2 ± 0.7), and UDCA (3.4 ± 0.8) were notably decreased, respectively (*P. dorei*, *p* = 0.0111; *L. lactis*, *p* = 0.0023; UDCA, *p* = 0.005). Furthermore, the endotoxin level in mouse serum was significantly reduced in the *P. dorei* group. Serum AST and bilirubin levels were significantly improved in the *P. dorei*-treated group (Figure 2E), whereas ALT levels showed only a non-significant trend toward improvement (Fig. S1).

We evaluated mRNA expression of the cytokines and chemokines in mouse liver (Figure 2F). The gene expression of cytokine TNF-α, IL-1β, IL-6, and IFN-γ revealed elevated in the Western group compared to the normal group. *P. dorei* group showed downregulated levels of the cytokines. The mRNA expression of chemokines, which play a pivotal role in MASLD pathophysiology, significantly decreased in the *P. dorei* group compared with the Western group. The lipid metabolism genes including the sterol regulatory element binding protein 1c (SREBP-1c) and fatty acid synthesis (FAS) were increased in the Western group compared to the normal diet group, but there was no significant change in the *P. dorei*, *L. lactis* and UDCA group. Nevertheless, lipid beta oxidation-related genes, including peroxisome proliferator-activated receptor α (PPAR-α) increased in *P. dorei* group. These results suggest that the administration of *P. dorei* alleviates lipid accumulation and inhibits inflammation response of the fatty liver to hepatitis progression.

### Gut microbiota and liver RNA transcription are changed by P. dorei oral administration

**Figure 3.**
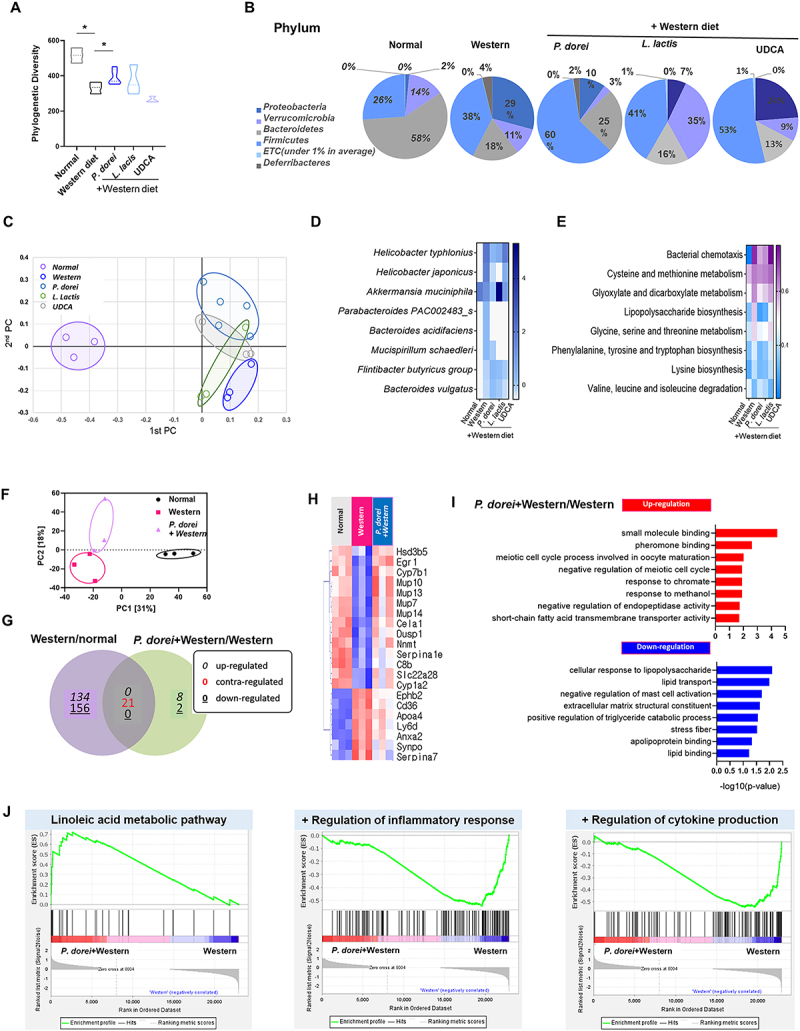
Gut microbiota and liver RNA transcription are changed by *P. dorei* administration. Stool samples and liver tissues were collected from mice to perform 16S rRNA and RNA-Seq analysis. (A) Phylogenetic diversity based on species richness in cecal samples (*n* = 3–5 per group). (B) Phylum-level microbial composition in cecal samples from NC, WD, *P. dorei. L. lactis*, and UDCA. Abundance and relative abundance (%) of the two major phyla. (C) Beta diversity was plotted as a PCoA plot showing the similarity of bacterial community structure based on Bray Curtis (D) Heatmap analysis for significantly different species. (E) Comparative analysis of the estimated functional profiles based on KEGG orthology in different experimental group. (F) Expression similarity between samples by principal component analysis. (G) Venn diagram showing specific or common differentially expressed genes between Western/normal and *P. dorei*+Western/Western (*n* = 3 per group). (H) Heatmap showing genes commonly up- or down-expressed (*n* = 3 per group). (I) Bar chart of genes involved in GO biological processes up- and down-regulated by *P. dorei* administration Gene set enrichment analysis for KEGG items (*n* = 3 per group). (j) Gene set enrichment analysis for KEGG items. Data are expressed as the means with SEM. *p < 0.05, **p < 0.01.

To investigate the role of gut microbiota in mediating MASH induced by a Western diet and the administration of specific strains, we evaluated the gut microbiome using the 16S-based Microbial Taxonomic Profiling platform provided by EzBioCloud. The Jackknife-based alpha diversity was higher in the *P. dorei* group compared to the Western group (Western: 332 ± 27; *P. dorei*: 391 ± 43) (Figure 3A). Beta diversity analysis, examining associations between distance measurements and taxonomic profiles, demonstrated that replicates within the same group clustered closely, while different groups were clearly separated (Figure 3B). At the phylum level, the relative abundance of *Bacteroides* was notably higher in the *P. dorei* group (25%) compared to the Western group (18%) (Figure 3C,D). Notably, species-level microbial diversity was also elevated in the *P. dorei* group compared to other groups (Figure 3E).

To examine the role of *P. dorei* in mitigating inflammation and lipid accumulation in mice subjected to a Western diet, we conducted RNA-seq analysis of liver tissues and generated transcriptomic profiles. Significant results were defined by a fold change greater than 2, normalized data (log2) exceeding 1, and a p-value below 0.05, with adjustments made for False Discovery Rate (FDR). Heatmap analysis of differentially expressed genes (DEGs) revealed marked upregulation and downregulation patterns in the *P. dorei* group compared to the Western diet group. Principal Component Analysis (PCA) demonstrated clear separation among the three experimental groups (Figure 3F). Correlation analysis of gene expression was statistically evaluated and visualized, identifying a total of 21 genes inversely regulated by *P. dorei* (Figure 3G). The dendrograms illustrating gene expression similarities among samples displayed distinct differences between the Western diet and *P. dorei* groups (Figure 3H). Additionally, we performed Gene Ontology (GO) enrichment analysis focusing on biological processes, which revealed that pathways related to short-chain fatty acids (SCFAs), lipid metabolism, immune response, and general metabolic processes were modulated in the *P. dorei* group (Figure 3I). Furthermore, gene set enrichment analysis (GSEA) based on microbial taxonomic profiles identified several significantly enriched pathways in the *P. dorei* group compared to the Western diet group. Among them, linoleic acid metabolism was notably enriched, suggesting a potential reprogramming in microbial function related to fatty acid processing and host – microbiome metabolic interaction (Figure 3J). Linoleic acid plays a complex role in hepatic lipid metabolism and inflammation. Dysregulated linoleic acid metabolism has been associated with the progression of MASLD, particularly during the transition to steatohepatitis.^25^ Certain gut bacteria possess enzymes such as linoleate isomerase and hydratase, which convert linoleic acid into conjugated linoleic acids and other bioactive lipids.^26^ These microbial metabolites may exert hepatoprotective effects by reducing lipid accumulation and attenuating pro-inflammatory signaling.

## Metabolic profiles were changed by *P. dorei* oral administration

**Figure 4.**
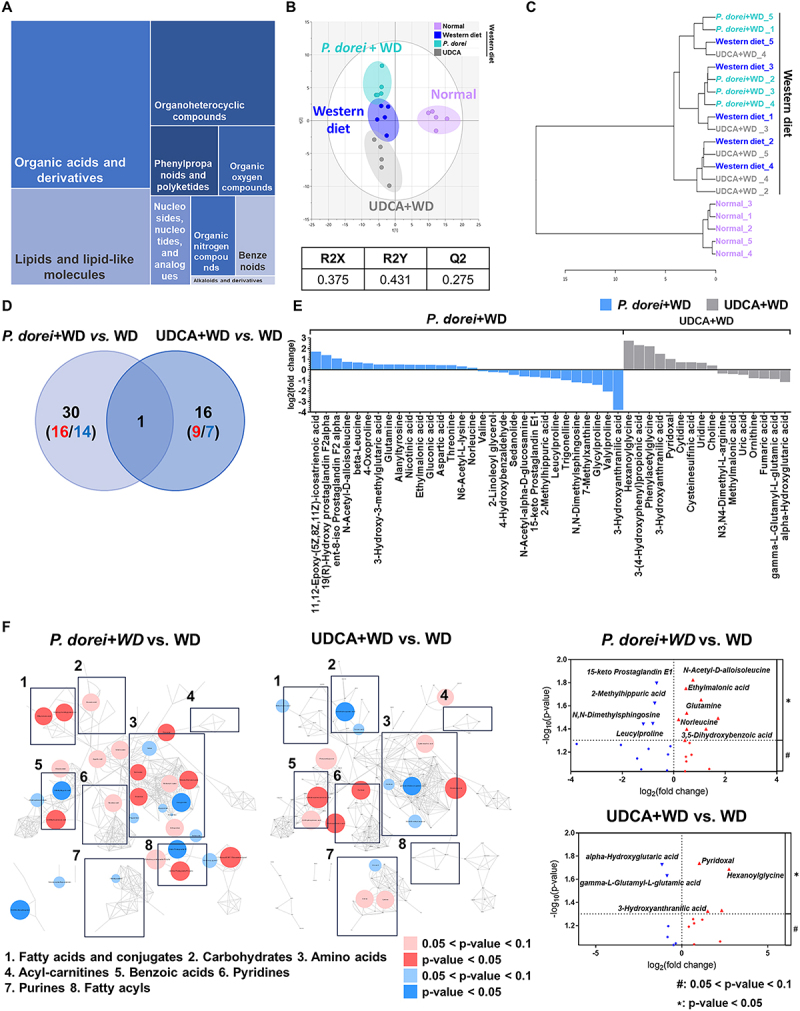
Differential metabolic signatures in mouse cecum across diet and administration groups. (A) Chemical classification of identified metabolites in mouse caecum provided by HMDB (http://www.hmdb.ca). The 176 compounds (96.7%) are categorized into nine superclasses. (B) The score scatter plot of 182 cecal metabolites by partial least squares-discriminant analysis (PLS-DA). (C) Dendrogram of hierarchical cluster analysis of cecum metabolomics data from Western diet group (*n* = 5), administration group (*P. dorei* administration group, *n* = 5; UDCA administration group, *n* = 5), and normal chow diet (*n* = 5) group. Each sample on the y-axis reflects one cecum sample. The x-axis shows the similarity levels expressed as Pearson distances. Horizontal and vertical lines depict differences and clustering of samples in the distances, respectively. (D) The metabolites show specific abundance patterns in other groups compared to the Western diet group. (E) The bar plots show the fold-change of group-specific metabolites in the log_2_ scale compared to the Western diet (*p* < 0.05). (F) The network is constructed based on chemical structural similarity (Tanimoto score) and KEGG reaction pair (substrate-product relation), which results in distinctive metabolic modules indicated by the box. Red and blue colors present significantly higher or lower abundance in *P. dorei*, and UDCA groups, respectively, compared to the Western diet (Student’s *t*-test; *p* < 0.05: red, blue; *p* < 0.1: pink, sky-blue). The node sizes are determined by the ratios. Volcano plot for identification of metabolites with significant differences in the *P. dorei*, and UDCA, respectively, compared to the Western diet group. The X-axis presents the fold change in the log_2_ scale, and the Y-axis indicates the statistical significance (value of *P*) in the log_10_ scale based on the student’s *t*-test. Red and blue colors present significantly higher or lower abundance in other groups, respectively, compared to the Western diet (*p* < 0.05; 0.05< *p* < 0.1).

The metabolic profiles of the cecal contents were obtained from groups subjected to normal chow diet, Western diet, and administration of *P. dorei* and UDCA. A total of 181 metabolites were identified and semi-quantified using both SCFAs-targeted and untargeted analysis. Among them, 176 metabolites were classified based on the chemical ontology analysis as following superclass levels: organic acids and derivatives (33.5%), lipids and lipid-like molecules (19.3%), organoheterocyclic compounds (18.8%), and phenylpropanoids and polyketides (6.8%) (Figure 4A). Similar to the microbial taxonomic profiles, the metabolic profiles were mainly discriminated between the normal diet group and the other groups using partial least squares discriminant analysis (Figure 4B). Likewise, the dendrogram showed the metabolic profiles of the normal diet group were distinctively clustered from the Western diet and administration groups (Figure 4C).

First, we explored the metabolites, showing treatment-specific changes (*P. dorei* and UDCA groups), compared to the Western diet group. The number of metabolic features specifically altered in *P. dorei* group was higher than the number in UDCA group (Figure 4D). Thirty-one metabolites were specifically changed in the *P. dorei* group, of which 16 and 14 compounds were significantly up- and downregulated, respectively (*p* < 0.1) (Figure 4E). 16 metabolites showed UDCA treatment-specific changes. Common change was determined only in 3,5-dihydroxybenzoic acid, which was significantly up-regulated in both treatments. (Figure 4F,G).

To effectively identify key metabolic modules, an integrated metabolic network analysis was applied, built upon chemical structural similarity (*Tanimoto* score) and enzymatic reaction connectivity (KEGG reaction pair)(Figure 4D).^23^ The *P. dorei* administration group showed the distinctive changes in such modules as fatty acids-conjugates, amino acids, benzoic acids, and fatty acyls. In particular, the *P. dorei* group showed substantial increases (*p* < 0.05) in amino acids (e.g., norleucine, threonine) and fatty acid acyls (e.g., ethylmalonic acid, 3-hydroxy-3-methylglutaric acid). On the contrary, the UDCA group was characterized by the significant down-regulation in carbohydrate and up-regulation in pyridine modules.

**Figure 5.**
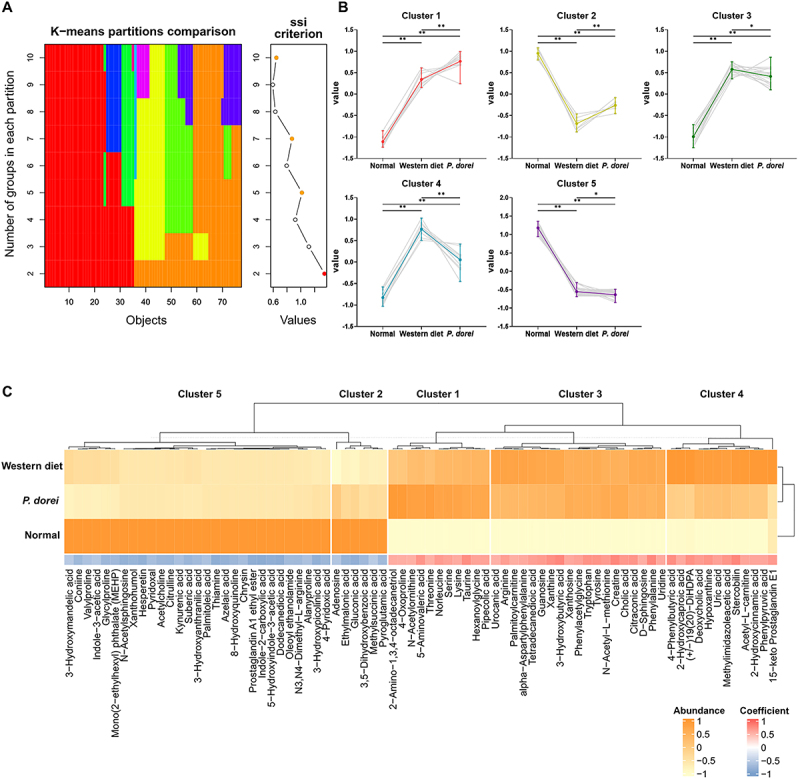
Evaluating group-specific metabolites across diet and administration. (A) An appropriate number of K-means clusters was determined based on simple structure index (SSI) in the median values of metabolic features on the normal chow diet, *P. dorei* administration, and Western diet group. (B) The abundance patterns of metabolic features were grouped into five clusters. (C) The abundance and correlation patterns of individual metabolites were visualized on a heatmap (normal chow diet: 0, *P. dorei* administration: 1, and the Western diet: 2).

Next, we examined how the metabolites changes by the treatments were associated with improved patho-phenotypes (e.g., AST, TBIL), simultaneously comparing the levels observed in normal chow diet and Western diet groups. This analysis allowed for the interpretation of the metabolic changes: If the levels were similar to those in the normal diet group but different from those in the Western diet group, the effect was considered a normalized effect. Accordingly, we identified 77 metabolites (42.3%), showing group-specific patterns among three groups (normal diet, Western diet, *P. dorei*) (Fig. S2). Subsequently, K-means clustering analysis identified five clusters (Figure 5A, Fig. S3).

Among them, cluster 2 (*n* = 6) and 4 (*n* = 12) consisted of the metabolites, potentially retaining the normalized effects by the treatment (Figure 5B, Fig. S4). Cluster 2 included adenosine, fatty acids (ethylmalonic acid and methylsuccinic acid), and gluconic acid, showing significant up-regulation patterns in both normal diet and *P. dorei* group, relative to Western diet group. On the contrary, cluster 4 members, including purines (hypoxanthine, uric acid), fatty acyls, and deoxycholic acid presented significant down-regulation patterns (Figure 5C).

### P. dorei on the STAM mouse model

**Figure 6.**
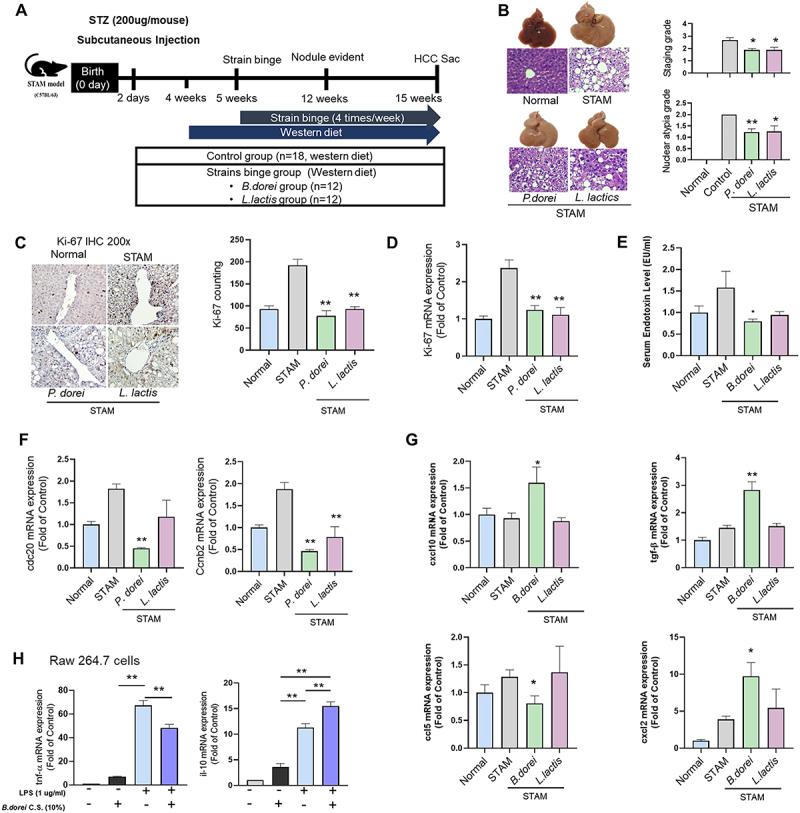
*P. dorei* inhibits nuclear atypia in the STAM mouse model. (A) Scheme of the animal experiment. (B) Representative H&E stained liver sections. Atypia grade and staging grade. (C) IHC with Ki-67 (D) mRNA expression of Ki-67. (E) Serum endotoxin level. (F) Proliferation markers (cdc20 and Ccnb2) analysis (G) Expression of chemokines and cytokines (h) in-vitro analysis for antififlammatory effect of *P. dorei*. All data are expressed as the means with SEM. *p < 0.05, **p < 0.01.

We investigated the role of *P. dorei* in liver tumorigenesis using STAM mouse model (Figure 6A). STAM mouse, a MASH-cirrhosis-hepatocarcinogenic model, offers the advantage of monitoring the disease progression in a controllable method. In our study, the STAM group induced fatty liver and nuclear atypia but not complete HCC. Administration of *P. dorei* decreased nuclear atypia of hepatocytes in STAM mouse model. Nuclear atypia, which known to pathological feature of tumor cells, has been used as a pathological marker for HCC.^23^ Considered as an indicator of malignancy, nuclear atypia was clearly decreased in *P. dorei* and *L. lactis* group (*p* < 0.01 and *p* = 0.01, respectively) (Figure 6B). Inflammation grade was significantly reduced in the *L. lactis* group (*p* < 0.01), and staging grade decreased in both *P. dorei* and *L. lactis* groups (*p* = 0.01). Body weight and liver weight were similar in the strain groups and the STAM group.

Administration of *P. dorei* prevented the proliferation of hepatocytes (Figure 6C). The Ki-67 mRNA expression of hepatocytes was increased in the STAM group, while decreased in the *P. dorei* and *L. lactis* groups significantly (Figure 6D). Furthermore, the endotoxin level in mouse serum was significantly reduced in the *P. dorei* group (Figure 6E). We analyzed the mRNA expression of Cyclin B2 (CCNB2) and cell division cycle 20 (CDC20) genes in the mouse liver, the *P. dorei* group downregulated the genes significantly compared to the STAM group. These data indicate that *P. dorei* plays a vital role in tumorigenesis by inhibiting CDC20 and CCNB2 (Figure 6F). Furthermore, administration of *P. dorei* showed antitumor effects by the inflammatory response during tumorigenesis (Figure 6G). In the liver mRNA expression, the transforming growth factor-β (TGF-β), CXCL2, and CXCL10 levels were noticeably increased in the *P. dorei* group compared with the STAM group. CCL5, extracellular matrix protein 1 (ECM1), was decreased in *P. dorei* group. In vitro analysis, *P. dorei* significantly decreased inflammatory response (Figure 6H). These results further show that *P. dorei* has anti-tumor and anti-inflammation roles.

### Phocaeicola dorei-driven metabolic activity leads to hepatic inflammation

**Figure 7.**
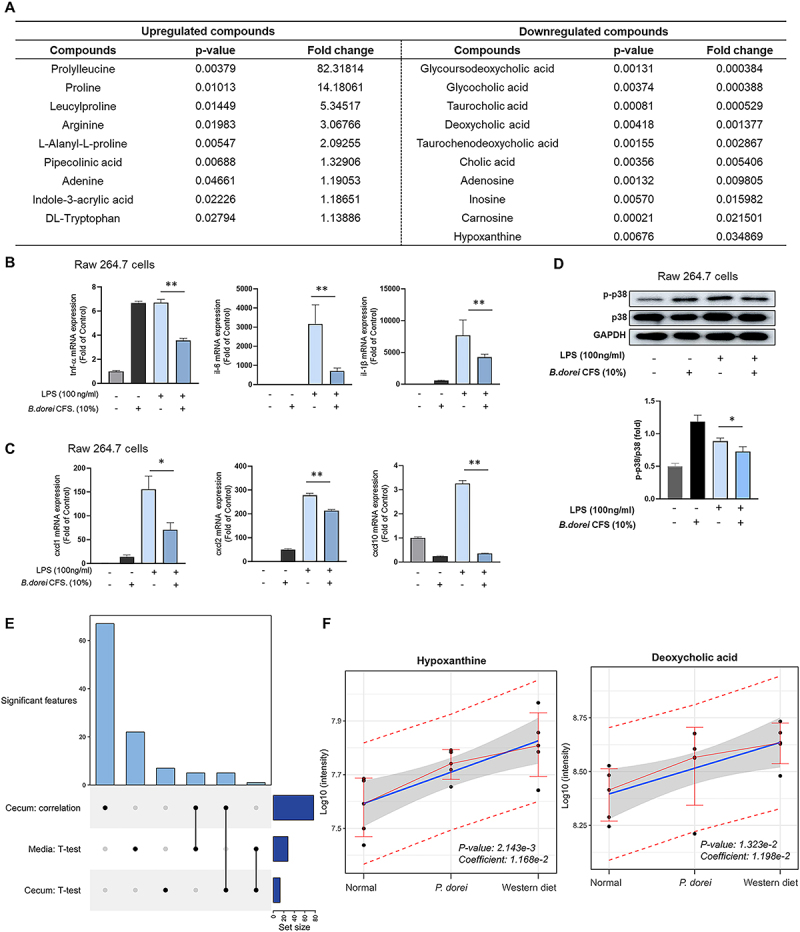
*P. dorei* CFS reduces LPS-induced inflammation in Raw 264.7 cells. (A) Metabolomic comparison between RCM control and cell-free supernatant of *P. dorei*. (B-D) expression of proinflammatory cytokines, chemokines genes, and p-p38. (E) The significant features were identified in the comparison of the Western diet and *P. dorei* diet within the cecum and media. (F) Relative abundance of metabolites (e.g., deoxycholic acid, hypoxanthine) in cecum samples. Statistical significance in linear regression is determined based on *p* < 0.05.

We examined whether the hepatic inflammation was directly inhibited by *P. dorei*-associated metabolic activity (e.g., production of beneficial compounds or depletion of metabolites with adverse effects in this experimental setting). Extracellular metabolites produced by *P. dorei* (CFS) were profiled and comparatively analyzed with RCM control (student’s t-test, *p* < 0.05) (Figure 7A). Tryptophan metabolites (tryptophan, indole-3-acrylic acid) and amino acids showed substantially higher in the *P. dorei* CFS, than in RCM control. On the contrary, bile acids exhibited dramatically lower abundance compared to RCM control, including glycoursodeoxycholic acid (GUDCA), glycocholic acid (GCA), taurocholic acid (TCDCA), deoxycholic acid (DCA), and cholic acid (CA).

Moreover, *P. dorei* CFS downregulated the inflammatory cytokine and chemokines in LPS-induced 264.7 Raw cells (Figure 7B). The LPS-induced cells treated with *P. dorei* CFS were reduced the gene expression level of TNF-α, CXCL1, and CXCL2 compared with LPS-stimulated cells (*p* < 0.0001; *p* = 0.0432; *p* = 0.0003, respectively). Especially, the CFS strongly reduced the mRNA expression of CXCL10 about 9-fold changes (*p* < 0.0001) (Figure 7C). These results indicated that *P. dorei* CFS prevents the synthesis of pro-inflammatory cytokines and chemokine in activated macrophages. *P. dorei* CFS reduces LPS-induced inflammation in Raw 264.7 cells (Figure 7D).

We compared shared metabolites from both cecal content and CFS to identify metabolites potentially linked to the effect of *P. dorei* treatment on liver injury (Figure 7E). Among the 77 cecal metabolites and 30 extracellular metabolites from *P. dorei* CFS, five metabolites were found in common: adenosine, cholic acid, deoxycholic acid, mono(2-ethylhexyl) phthalate, hypoxanthine. Particularly, bile acids (deoxycholic acid, colic acid) and hypoxanthine depleted in *P. dorei-*culture media, showed decreased pattern in both *P. dorei* treatment and normal diet groups, compared to Western diet groups (Figure 7F and Fig. S3). These findings suggest that the high consumption rate of specific bile acids and hypoxanthine by *P. dorei* may serve as underlying mode-of-action for anti-inflammatory process.

**Figure 8.**
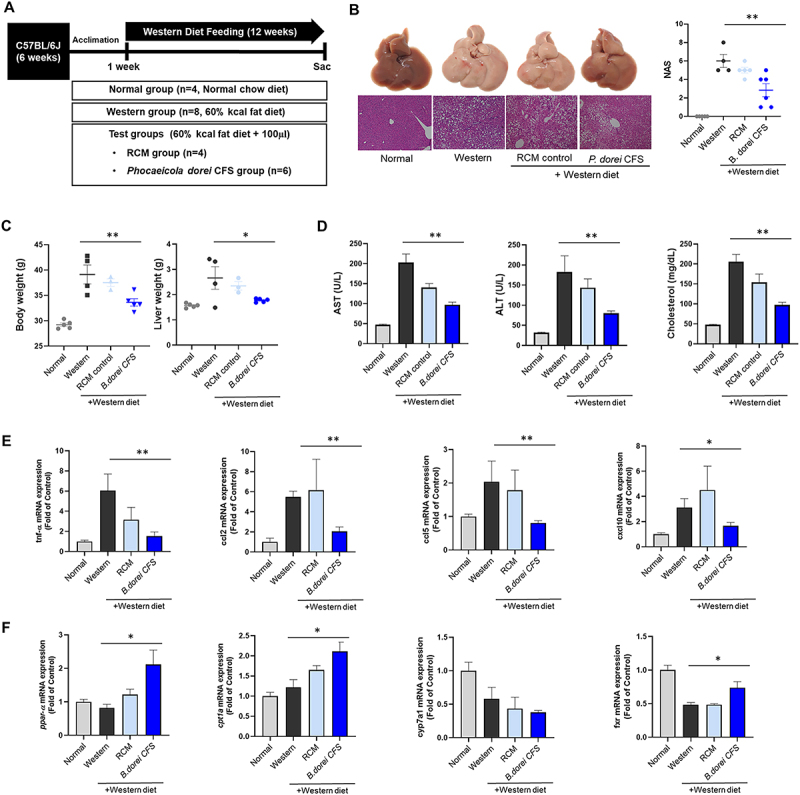
*P. dorei* CFS attenuates hepatic injury in the Western diet-induced MASH mouse model. (A) Scheme of the animal experiment. (B) Representative images on H&E-stained liver tissues, and comparison of NAS. (C) Change of weight. (D) Liver enzyme analysis. (E) Expression of proinflammatory cytokines and chemokines genes. (F) Expression of lipid metabolism markers. All data are expressed as the means with SEM. *p < 0.05, **p < 0.01.

We investigated the role of potential *P. dorei* CFS in the Western diet-induced MASH mouse model (Figure 8A). In the NAS, the Western diet group was significantly increased compared with the normal diet group (*p* < 0.001), and the *P. dorei* CFS group markedly decreased compared with the Western diet group (*p* = 0.0025) (Figure 8B). The body weight and liver weight were significantly decreased in *P. dorei* CFS compared with the Western group (*p* < 0.0001) (Figure 8C)., but there was no significant difference with the growth medium control group (RCM). Moreover, blood biochemical measurements, including AST, ALT, and cholesterol levels, were markedly decreased in both RCM and *P. dorei* CFS groups (Figure 8D). The TNF-α mRNA expression reduced about 3-fold in the *P. dorei* CFS group compared with the Western group (*p* = 0.0044). However, the *P. dorei* CFS group could not change the mRNA expression of chemokines, including CC motif chemokine ligand 2 (CCL2), CC motif chemokine ligand 5 (CCL5), and CXCL10 in the liver (Figure 8E). Additional mRNA expression of lipid β-oxidation gens, PPAR-α and c, significantly increased in *P. dorei* CFS group compared with the Western group (*p* = 0.0207; *p* = 0.0100). The expression of bile acid synthesis enzymes, CYP7a1, and farnesoid X receptor (FXR) showed no statistical change in the *P. dorei* CFS group compared with the Western and the RCM group (Figure 8F). These results suggest that the inflammatory response is improved by inducing beta-oxidation of fatty acids and is related to FXR signaling.

## Discussion

Gut dysbiosis contributes to the pathogenesis of obesity-related disorders, including metabolic syndrome and MASLD.^27^ Since MASLD progression was robustly associated with metabolic dysregulations, modulating the gut microbiota to a healthy state can be effective in the treatment of MASLD.^6,8,28^ We accomplished an analysis of the human stool microbiome and found that the relative abundance of *P. dorei* is higher in patients with hepatitis than in the normal population. Given previous reports that *Phocaeicola* spp. can modulate inflammatory pathways and bile acid metabolism,^29^ its proliferation might serve as an attempt to restore intestinal-liver axis homeostasis. Considering that *Phocaeicola* has an important role in maintaining a healthy gut ecosystem, we hypothesized increasing *P. dorei* can provide a benefit by serving as a compensatory role for the progression of MASLD.

Inflammatory processes are crucial in the potential progression of MASLD. Cytokines and chemokines might also play a pivotal role in MASLD pathophysiology.^30^ Expression of chemokines and chemokine receptors has also been shown to be upregulated in the liver of obese patients with severe steatosis and MASH.^31^ In this study, In this study, *P. dorei* inhibited the expression of toll like receptor-associated inflammatory cytokines and chemokines in animal and cell-line models. Another study demonstrated that *P. dorei* reduced gut microbial LPS production and inhibited inflammation-related atherosclerosis.^14^ In addition, *P. dorei* improved acute colitis by regulating bile salt hydrolase activity and the FXR and proinflammatory cytokines pyrin domain-containing 3 signaling pathway. Taken together, *P. dorei* is a promising probiotic to improve the inflammation in MASLD.^32^

In our results, *P. dorei* oral administration activated lipid beta-oxidation and inhibited inflammation response of the fatty liver to hepatitis progression. Previous report suggested that oral administration with probiotic had a positive effect on lipid profiles and inflammatory cytokines in patients with MASLD.^33^ Regarding *P. dorei*, another study demonstrated its superior cholesterol-lowering capabilities and probiotic properties. These findings suggest that *P. dorei* can be a promising next generation-probiotics for improving lipid profiles.

PPAR-α decreases lipid storage by FA transport and β-oxidation and reduces inflammatory responses in liver tissue by regulating gluconeogenesis and amino acid metabolism.^4^ In our MASH model, expression of PPAR-α and CPT1α, which promotes lipid beta-oxidation, increased in the *P. dorei* CFS group. These results showed that *P. dorei* CFS increased PPAR-α and CPT1α, thereby regulating FA metabolism and inflammatory responses in the liver tissue.

The hepatocarcinogenesis is a complex multistep biological process and several signaling pathways lead to dis-regulated and uncontrolled cell division and metastasis.^34^ In our results, expressions of the Ki-67 marker were significantly reduced in the *P. dorei* and *L. lactis* groups compared with the STAM control group. Additionally, TGF-β expression was increased in *P. dorei* group. TGF-β signaling participates in all stages of disease progression, from initial hepatic injury through inflammation to cirrhosis and cancer.^35^ Moreover, TGF-β has been reported to act as a tumor suppressor in normal cells, promoting cellular differentiation and apoptosis and inhibiting cellular proliferation. Also, CXCL10 has been reported to modulate anti-tumor immunity in certain cancers, as opposed to being upregulated in MASH.^36^ Therefore, our data suggest that *P. dorei* may ameliorate the tumor progression by promoting the TGF-β and CXCL10 which modulate anti-tumor immunity.

Recent studies demonstrated that modulating the gut microbiome and its metabolites through probiotic administration is a promising approach for MASLD improvement by regulating the gut-liver axis.^8,37,38^ To further explore the functional implications of microbiota alterations, we performed gene set enrichment analysis using the 16S-based microbial taxonomic profiles. This analysis identified several enriched pathways in the *P. dorei* group, including linoleic acid metabolism. Although linoleic acid levels were not directly measured in our metabolomic dataset, previous studies have suggested that gut microbes bio-transform linoleic acids and produce its conjugated forms and down-stream products, which may activate receptors such as GPR40 and GRP120, modulating host lipid signaling and inflammation.^39^ Given the role of linoleic acid – derived mediators, the observed enrichment in microbial linoleic acid metabolism may represent a beneficial remodeling of microbial functionality.

To explore specific microbial metabolites that may play a direct role in host inflammation, we performed untargeted metabolomic profiling of *P. dorei*-cell free supernatant. First, we screened 77 metabolites based on clustering analysis, identifying similar patterns between *P. dorei* treatment group and normal diet group, compared to the Western diet group. Next, the comparative analysis revealed significantly higher levels of tryptophan metabolites and amino acids, and notably lower levels of bile acids in *P. dorei* CFS compared to RCM control.

A previous study reported that a high-fat diet caused oxidative stress and resulted in changes in purine metabolism and hypoxanthine accumulation, leading to lipid accumulation in hepatocytes and the development of MASLD. In addition, Western diet has been reported to increase biliary secretion and fecal excretion of BAs, especially secondary bile acids, deoxycholic acid (DCA) and lithocholic acid (LCA).^40,41^ Another report demonstrated Western diet impaired BA transport in colon and reduced FXR activity, which suppressed the activity of NF-kB related inflammation factors.^42^ Consistent with the previous studies, our current results showed the lower levels of hypoxanthine and deoxycholic acid in normal diet group compared to the Western diet group. In addition, *P. dorei* treatment reduced hypoxanthine and deoxycholic acid levels similar to those in the normal group, suggesting that hypoxanthine and deoxycholic acid reduction could serve as an effector or mediator of the improvement of fatty liver disease and inflammation by *P. dorei* treatment.

Therefore, we evaluated whether the *P. dorei* metabolites exhibit an anti-inflammatory effect in MASH. As a result, the *P. dorei* CFS dramatically decreased TNF-α and CXCL10 in both *in vitro* and *in vivo* model experiments. CXCL10 is a pivotal molecule promoting the transition from steatohepatitis to progressively hepatocellular injury and inflammation in steatohepatitis.^36^ Therefore, the *P. dorei* CFS can inhibit the progression to steatohepatitis by regulating the expression of proinflammatory molecules, including CXCL10. Further investigation is needed to determine whether the significant metabolites derived from *P. dorei* show consistent patterns between preclinical and clinical studies within the context of microbiome-based therapeutics.

Given the global burden of MASLD and the lack of approved pharmacological therapies, microbial approaches hold great therapeutic potential. While growing evidence has implicated gut microbiota in the progression of MASLD, few studies have specifically addressed the mechanistic roles of gut commensal bacteria species. *P. dorei* has emerged as a candidate with immunomodulatory effects, yet its direct impact on MASLD progression remains underexplored. By integrating human clinical data with experimental validation in animal models, this study provides crucial insights into the role of *P. dorei* in ameliorating hepatic inflammation and lipid accumulation.

In our result, serum AST and bilirubin levels were reduced in the *P. dorei*-treated group. However, ALT, which better reflects hepatocellular damage, showed no significant change. Since AST is also found in muscle and bilirubin can rise due to hemolysis or cholestasis, these markers alone may not reliably indicate liver-specific effects. This point was added to provide the limitations of serum-based interpretation and to emphasize the importance of integrating histology in evaluating hepatic outcomes.

In conclusion, *P. dorei* and its metabolites ameliorate MASLD progression by modulating bile acid pool, lipid accumulation, inflammation, and tumor cell proliferation. Therefore, *P. dorei* could be a promising candidate for a new therapeutical approach to MASLD. Further studies are required to elucidate the underlying mechanisms and develop effective strategies for MASLD treatment.

## Supplementary Material

Supplementary file.docx

## Data Availability

All data generated during this study are included in this article. All 16S rRNA sequences were deposited in the EzBioCloud Microbiota database and the NCBI Short Read Archive under the bioproject number PRJNA532302 (https://www.ncbi.nlm.nih.gov/bioproject/?term=PRJNA532302).

## List of abbreviations

3NPH: 3-Nitrophenylhydrazine Hydrochloride
ALT: Alanine Transaminase
AST: Aspartate Transaminase
BMI: Body Mass Index
CA: Cholic Acid
CCL5: CC Motif Chemokine Ligand 5
CCNB2: Cyclin B2
CDC20: Cell Division Cycle 20
CFS: Cell-Free Supernatant
CPT-1A: Carnitine O-Palmitoyltransferase 1
DCA: Deoxycholic Acid
DEGs: Differentially Expressed Genes
ECL: Enhanced Chemiluminescence
ECM1: Extracellular Matrix Protein 1
FAS: Fatty Acid Synthesis
FXR: Farnesoid X Receptor
GCA: Glycocholic acid
GGT: Gamma-glutamyl Transferase
GO: Gene Ontology
GSEA: Gene Set Enrichment analysis
GUDCA: Glycoursodeoxycholic Acid
HDL: High-density Lipoprotein
HCC: Hepatocellular Carcinoma
LC: Liquid Chromatography
LCA: Lithocholic Acid
LPS: Lipopolysaccharides
MASLD: Metabolic Dysfunction-associated Steatotic Liver Disease
MS: Mass Spectrometry
NAS: Non-Alcoholic Fatty Liver Disease Activity Score
PCA: Principal Component Analysis
PPAR-α: Peroxisome Proliferator-activated Receptor α
RCM: Reinforced Clostridial Medium
SCFAs: Short-chain Fatty Acids
SREBP-1c: Sterol Regulatory Element Binding Protein 1c
TCDCA: Taurochenodeoxycholic Acid
TG: Triglycerides
TGF-β: Transforming Growth Factor-β
UDCA: Ursodeoxycholic Acid
