# Supplementary material for: Phocaeicola dorei ameliorates progression of steatotic liver disease by regulating bile acid, lipid, inflammation and proliferation
Source: Gut Microbes. 2025 Aug 3;17(1):2539448. doi: 10.1080/19490976.2025.2539448 (PMC12323408; doi:10.1080/19490976.2025.2539448)
Supplement: Supplementary file.docx [file KGMI_A_2539448_SM2722.docx]

*Supplementary file.*

***Phocaeicola dorei* ameliorates progression of steatotic liver disease by regulating bile acid, lipid, inflammation and proliferation**

Jieun Choi^1†^, Ye Rin Choi^2†^, Min Kyo Jeong^2†^, Hyun Ho Song^1^, Jeong Seok Yu^1^, Seol Hui Song^2^, Jeong Ha Park^2^, Min Ju Kim^2^ Hyunjoon Park^2†^, Young Lim Ham^3^, Sang Hak Han^4^, Dong Joon Kim^2^, Do Yup Lee^1,5^*, Ki Tae Suk^2^*,

**Methods**

***Tissue and serum analysis***

Specimens were fixed with 10% formalin, embedded in paraffin, and the tissue sections were processed with hematoxylin and eosin. NAFLD activity score (NAS), an objective index for classifying the grade of fatty liver, was assessed.^1^ All biopsy specimens were analyzed by a hepato-pathologist (S. H. H.). Fatty liver was classified according to the MASH clinical research network scoring system for MASLD from grades 0 to 3 (0: <5%, 1: 5%–33%, 2: 34%–66%, and 3: >66% of steatosis). Inflammation was classified from grades 0 to 3 (0: none, 1: 1–2 foci per ×20, 2: 2–4 foci per ×20, and 3: >4 foci per ×20 field). Guidelines based on NAS may be useful in recognizing histological scoring systems that cover the full spectrum of MASLD. MASH was determined based on an overall pattern of histological hepatic injury consisting of lobular inflammation, steatosis, and ballooning ^2^. We also scored lobular inflammation, steatosis, and ballooning according to the MASLD activity scoring system.

Mouse serum samples were collected from 16-week-old controls and treated mice. Serum samples were obtained according to a previously described protocol. The collected serum samples were allowed to clot overnight at 2-8°C. After centrifugation for 2,000g for 20 minutes, the supernatant was collected and stored at -80°C. The serum endotoxin levels were determined using a limulus amoebocyte lysate assay (Lonza Walkersville, MD, USA) according to the manufacturer’s instructions.

***Quantification RT-PCR and Western blot***

Total RNA was extracted using the TRIzol Reagent (Thermo Fisher Scientific, MA, USA). RNA quality was determined using a spectrophotometer and was reverse transcribed using a high-capacity cDNA reverse transcription kit (Thermo Fisher Scientific, MA, USA), and qRT-PCR was performed using SYBR Green Master Mix (Thermo Fisher Scientific, MA, USA) in the Light Cycler 480 Real-Time PCR System.

Cells were incubated with RIPA buffer on ice for 30min and centrifuged at 16,000G, 4℃ for 20min. The supernatant was discarded, and only the pellet was collected. Proteins were resolved by 10% SDS-PAGE and transferred to nitrocellulose membranes. The blots were probed with the indicated primary antibodies phospho-p38, p-38 and GAPDH (Cell Signalling Technology, MA, USA) followed by incubation with the corresponding horseradish peroxidase-conjugated secondary antibodies (1:10,000 dilution). The membrane was reacted with the enhanced chemiluminescence (ECL) substrate solution and analyzed by Amersham Imager 680 (GE Healthcare, Buckinghamshire, UK).

***Cell culture and treatments***

Raw 264.7 cells were grown at 37 °C with 5 % CO_2_ in high-glucose Dulbecco’s modified Eagle’s medium (DMEM; Thermo Fisher Scientific, MA, USA), supplemented with 10 % heat-inactivated fetal bovine serum (Thermo Fisher Scientific, MA, USA) and 1 % Antibiotic-Antimycotic (X100) (Thermo Fisher Scientific, MA, USA). Cells (3 × 10^5^ cells/well) were seeded in a 12-well plate and incubated overnight. Then, cells were stimulated with LPS (100 ng/ml), treated with 10% of *P. dorei* culture supernatants, and incubated for 18h before total RNA isolation.

***Bacterial cultures and preparation***

The evaluated strain, *P. dorei,* was extracted from human feces and provided by Cheonlab Co., Ltd. Anaerobic pack (MGC A-04 Anaeropack), indicator (MGC A-66 Anaerobic indicator) and RCM (BD Difco Reinforced Clostridial Medium) medium for anaerobic bacterial culture were used in the experiment. The bacterial strains were identified based on the 16S rRNA gene sequence and cultivated in Reinforced Clostridial Medium (RCM; Thermo Fisher Scientific, MA, USA) for 24h at 35°C under anaerobic conditions prior to each experiment. Stock cultures were kept at -80°C in RCM containing 20% (v/v) sterile glycerol. *P. dorei* was grown in RCM broth for 24h at 35°C, then separated bacterial cell-free supernatant (CFS) by centrifugation at 4,000 rpm for 10 min at 4°C. The CFS was filtered through polyethersulfone syringe filters with a pore size of 0.45μm (Satorius, Goettingen, Germany). All samples were stored at −80°C.

***Immunohistochemistry***

Liver tissues were fixed in 10% formalin for 12h at room temperature, embedded in paraffin, and cut into 4-um sections. The tissue sections were then used to generate tissue microarray cores (1.5-mm diameter). The microarrays were deparaffinized in xylene I for 15 min and xylene II for 15 min at room temperature and were rehydrated in a graded ethanol series (100, 95, 80, and 75% ethanol, 5 min each). Subsequently, the microarrays were incubated with 3% H_2_O_2_ for 30 min at 37°C and 5% goat serum (Origene Technologies, Inc.) for 15 min at 37°C to block non-specific binding. The microarrays were then incubated with a monoclonal anti-Ki-67 antibody (1:2,000; cat. no. 12202S; Cell Signaling) at 4°C overnight. Subsequently, the sections were incubated with a secondary biotin-labeled IgG antibody (1:100; cat. no. SAP-9100; Origene Technologies, Inc.) at 37°C for 30 min. These sections were counterstained with Harris hematoxylin. From Ki-67 immunohistochemical stain, the findings were checked each on an average field of X200 and X400 as the area of stained cells using an imaging tool.

***Gut microbiota analysis***

Human feces were stored at -20°C as soon as the patient received 2-3 g of feces and moved to -80°C within one day. Genomic DNA was extracted with a QIAamp stool kit (Qiagen, Hilden, Germany) and amplified with the V3-V4 region of the bacterial 16S rRNA gene and dual-index barcodes. Purification of the amplicons was performed with an AMPure XP system (Beckman, CA, USA) and quantification of the purified amplicons was conducted using PicoGreen and quantitative PCR. After pooling the barcoded amplicons, sequencing was carried out using a MiSeq sequencer on the Illumina platform according to the manufacturer’s specifications.

Microbiota profiling was performed with the 16S-based Microbial Taxonomic Profiling platform of EzBioCloud (CJ Bioscience Inc., Republic of Korea). After taxonomic profiling of each sample, an EzBioCloud comparative analyzer was used. Taxonomic assignment of the reads was conducted with ChunLab’s 16S rRNA database (DB ver. PKSSU4.0).^3^ OTU picking was conducted with UCLUST and CDHIT with a 97% similarity cutoff. Subsequently, coverage, rarefaction, and alpha diversity indices were calculated. Beta diversity, including PCoA and UPGMA clustering, was shown in the comparative MTP analyzer. All 16S rRNA sequences were deposited in the EzBioCloud Microbiota database and the NCBI Short Read Archive under the bioproject number PRJNA532302.

***Quant-Seq microarray***

Total RNA was quantified with an Agilent 2100 bioanalyzer using the RNA 6000 Nano Chip (Agilent Technologies, Amstelveen, The Netherlands). RNA quantification was determined using an ND-2000 Spectrophotometer (Thermo Inc., DE, USA). The library was constructed using a QuantSeq 3′ mRNA-Seq Library Prep Kit (Lexogen, Inc., Austria) according to the manufacturer’s instructions. In brief, 500 ng of total RNA was prepared, an oligo-dT primer containing an Illumina-compatible sequence at its 5′ end was hybridized to the RNA, and reverse transcription was applied. After RNA template degradation, second-strand synthesis was initiated by a random primer containing an Illumina-compatible linker sequence at its 5′ end. The double-stranded library was purified using magnetic beads for removal of all reaction components. The library was amplified to add the complete adapter sequences required for cluster generation. The finished library was purified from PCR components. High-throughput sequencing was performed as single-end 75 sequencing using NextSeq 500 (Illumina, Inc., USA). For data analysis, QuantSeq 3′ mRNA-Seq reads were aligned using Bowtie2 (Langmead and Salzberg 2012). Bowtie2 indices were either generated from the genome assembly sequence or the representative transcript sequences for aligning to the genome and transcriptome. The alignment file was used for assembling transcripts, estimating their abundances, and detecting differential expression of genes. Differentially expressed genes were determined based on counts from unique and multiple alignments using coverage in Bedtools (Quinlan 2010). The Read Count data were processed based on the quantile normalization method using EdgeR within R using Bioconductor (Gentleman et al. 2004). Gene classification was based on searches in Database for Annotation, Visualization, and Integrated Discovery (DAVID, http://david.abcc.ncifcrf.gov) and Medline databases (http://www.ncbi.nlm.gov). A Porcine Quant-Seq microarray was performed using the customized service provided by eBiogen Inc, (Seoul, South Korea).

***Metabolite extraction***

The metabolic profiles of mouse cecal samples and bacterial cell-free supernatant (CFS) were obtained by liquid chromatography (LC)-mass spectrometry (MS). All mouse cecum samples (80 mg) were thawed under ice and combined with 1,100 µl of extraction solvent A (1:1 v/v of acetonitrile/water). The mixtures were vortexed until the cecum was uniformly suspended and centrifuged for 5 min at 13,200 rpm, 4 ℃. The supernatants (500 µl) were dispensed into the new tube (2 ml) for short-chain fatty acids (SCFAs) targeted analysis. Then, extraction solvent B (1:3 v/v of acetonitrile/methanol) was added 600 µl to the rest part of the supernatant. The mixtures were vortexed for 1 minute to proceed with the secondary extraction, followed by centrifugation at 13,200 rpm for 5 minutes. The supernatant (500 µl) was transferred to a new tube (1.5 ml) for untargeted metabolic profiling for LC-MS, ADDIN EN.CITE.DATA.

The CFS samples (100 µl) were mixed with 750 µl of extraction solvent C (3:3:2 v/v of methanol/iso-propanol/distilled water). The mixtures were sonicated for 10 minutes and centrifuged for 5 min at 13,200 rpm, 4 ℃. The supernatants (800 µl) were dispensed to a new tube (1.5 ml) for untargeted metabolic profiling for LC-MS. The supernatants were concentrated using a centrifugal vacuum concentrator (SCANVAC, Korea)(ADDIN EN.CITE.DATA.)

***Targeted analysis for short-chain fatty acids using LC-MS***

The supernatant (40 µl) dispensed from first extraction process was mixed with 20 µl of an EDC (1-ethyl-3-(3-dimethylaminopropyl) carbodiimide hydrochloride) at 120mM concentration in 6 % pyridine solution and 20 µl of a 200mM 3NPH (3-nitrophenylhydrazine hydrochloride) dissolved in 70 % acetonitrile. The mixture was reacted at 40 °C for 30 minutes and then added 1.92 ml of 70% acetonitrile.^4^

The derivatives were analysed by Thermo Q-Exactive Focus Orbitrap combined with an Vanquish UPLC system. Chromatographic separation was conducted through 2.1 x 150 mm ACQUITY BEH C18 with 1.7 μm particles column equipped with 5.0 x 2.1 mm BEH C18 VanGuard Pre-Column. The mobile phase included water (0.01 % formic acid) and acetonitrile (0.01 % formic acid). The gradient of LC was pre-set as follows: Equilibration in 15% buffer B for 2 min, 15-55 % buffer B gradient over 9 min, 100 % buffer B held for 1 min, and re-equilibration in 15 % buffer B for 3 min. The injection volume was 2 µl for both MS1 and MS/MS analysis. Mass spectra were acquired using Q-Exactive Focus Orbitrap (Thermo Fisher Scientific, Waltham, MA, USA) equipped with an electrospray ionization (ESI) interface (HESI-II) in negative ionization, and the system was controlled using Xcalibur 4.0 and Q-Exactive Tune software.

Raw data were processed by Tracefinder software (version 4.0, Thermo Fisher Scientific, San José, CA, USA). Mass tolerance for precursor ion and retention time tolerance was set to 5 ppm and 0.5 min, respectively

***Cecal metabolites profiling of LC-Orbitrap MS***

The dried extracts were reconstituted with 50 µl of 70% acetonitrile for LC-Orbitrap MS analysis. Chromatographic separation was performed using an Ultmate-3000 UPLC system (Thermo Fisher Scientific, Waltham, MA, USA) coupled with a 150 × 2.1 mm UPLC BEH 1.7 μm C18 column (Waters, Milford, MA, USA) and a 5.0 mm × 2.1 mm UPLC BEH 1.7 μm C18 VanGuard Pre-Column (Waters, Milford, MA, USA). The mobile phase consisted of buffer A (0.1% formic acid in water) and buffer B (0.1% formic acid in 100% acetonitrile). The flow rate was maintained at 0.35 ml/min with the following gradient profile: equilibration at 3% buffer B for 1 minute, a linear gradient from 3% to 100% buffer B over 9 minutes, 100% buffer B held for 1 minute, followed by re-equilibration at 3% buffer B for 3 minutes.

Mass spectrometry analysis was carried out using a Q-Exactive Plus Orbitrap instrument (Thermo Fisher Scientific, Waltham, MA, USA) operating in polarity-switching mode. Full MS scans were acquired within a mass range of 50-750 m/z at a resolution of 70,000 FWHM at m/z = 200, with an automatic gain control (AGC) target of 1e6 ions and a maximum injection time (IT) of 100 ms. Data-dependent MS/MS was performed on pooled samples for each ionization mode. The MS/MS settings were as follows: Top 5 MS1 ions; resolution, 17,500 at 200 m/z; AGC target, 1e5; maximum IT, 50 ms; isolation window, 1.0 m/z; normalized collision energy (NCE), 30; intensity threshold, 2e3 ions; apex trigger, 3-6 seconds; dynamic exclusion, 5 seconds. Inclusion lists were prepared with m/z values and retention times corresponding to bile acids, indoles, and trimethylamine-related compounds.

***Cultured media metabolites profiling of LC-Orbitrap MS***

Dried extracts were reconstituted with 50 µl of 80% methanol for LC-Orbitrap MS analysis. Chromatographic separation was performed using a Vanquish UPLC system (Thermo Fisher Scientific, Waltham, MA, USA) coupled with a 150 × 2.1 mm UPLC BEH 1.7 μm C18 column (Waters, Milford, MA, USA) and a 5.0 mm × 2.1 mm UPLC BEH 1.7 μm C18 VanGuard Pre-Column (Waters, Milford, MA, USA). The mobile phase was composed of buffer A (0.1% formic acid in water) and buffer B (0.1% formic acid in 100% acetonitrile). A flow rate of 0.35 ml/min was applied with the following gradient: equilibration at 10% buffer B for 2 minutes, increasing from 10% to 95% buffer B over 18 minutes, maintaining 95% buffer B for 5 minutes, and re-equilibrating at 10% buffer B for 5 minutes.

Mass spectrometric analysis was conducted using a Q-Exactive Focus Orbitrap (Thermo Fisher Scientific, Waltham, MA, USA) in polarity-switching mode. Full MS scans were performed over a mass range of 50-750 m/z with a resolution of 70,000 FWHM at m/z = 200, AGC target of 1e6 ions, and a maximum IT of 100 ms. Data-dependent MS/MS analysis was performed on pooled samples under each ionization mode. The parameters were: Top 3 MS1 ions; resolution, 17,500 at 200 m/z; AGC target, 5e4; maximum IT, 50 ms; isolation window, 2.0 m/z; NCE, 30; intensity threshold, 1e5 ions; apex trigger, 3-7 seconds; dynamic exclusion, 5 seconds.

**Supplementary Table 1. Characteristics of 129 Korean Patients with Liver Disease and 49 Healthy Controls.**

|  | **HC (n=49)** | | **MASLD (n=45)** | | **MASH (n=69)** | | **Cirrhosis (n=15)** | |
| --- | --- | --- | --- | --- | --- | --- | --- | --- |
| Age (years) | 61.2 | (7.8) | 59.8 | (8.2) | 54.3 | (14.0) | 69.8 | (8.38) |
| BMI (kg/m^2^) | 23.2 | (3.6) | 27.0 | (3.5) | 27.6 | (3.0) | 25.0 | (7.8) |
| AST (U/L) | 22.5 | (4.6) | 23.9 | (7.9) | 52.4 | (23.3) | 85.5 | (131.7) |
| ALT (U/L) | 18.7 | (7.1) | 25.6 | (10.7) | 67.0 | (34.8) | 54.6 | (73.8) |
| Creatine (mg/dL) | 0.9 | (0.2) | 0.9 | (0.2) | 1.4 | (4.3) | 0.9 | (0.2) |
| Cholesterol (mg/dL) | 174.3 | (39.0) | 177.9 | (37.5) | 179.6 | (38.3) | 135.1 | (79.8) |
| GGT (IU/L) | 26.2 | (17.6) | 40.0 | (37.0) | 67.7 | (54.1) | 165.1 | (239.8) |
| Triglyceride (mg/dL) | 118.7 | (108.3) | 163.8 | (121.2) | 171.9 | (144.3) | 85.4 | (51.0) |
| HDL (mg/dL) | 54.4 | (18.6) | 50.5 | (11.1) | 46.6 | (11.1) | 46.2 | (17.1) |

BMI, body mass index; AST, aspartate aminotransferase; ALT, alanine aminotransferase; GGT, gamma-glutamyl transferase; HDL, high-density lipoprotein


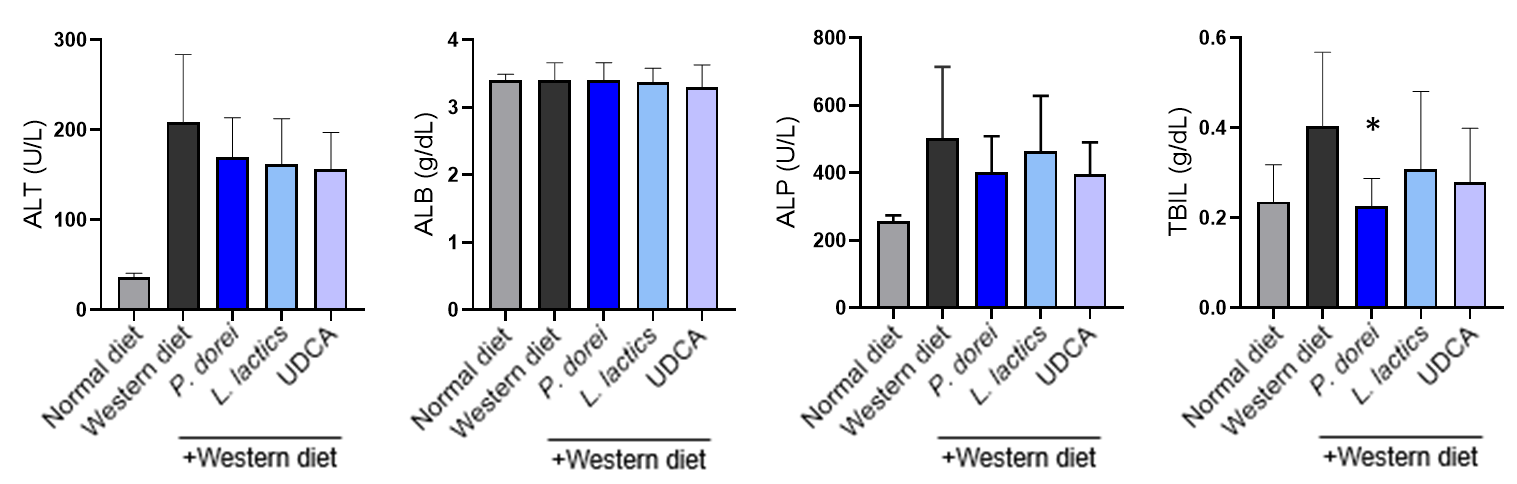


Supplementary Figure S1. Serum liver enzyme analysis


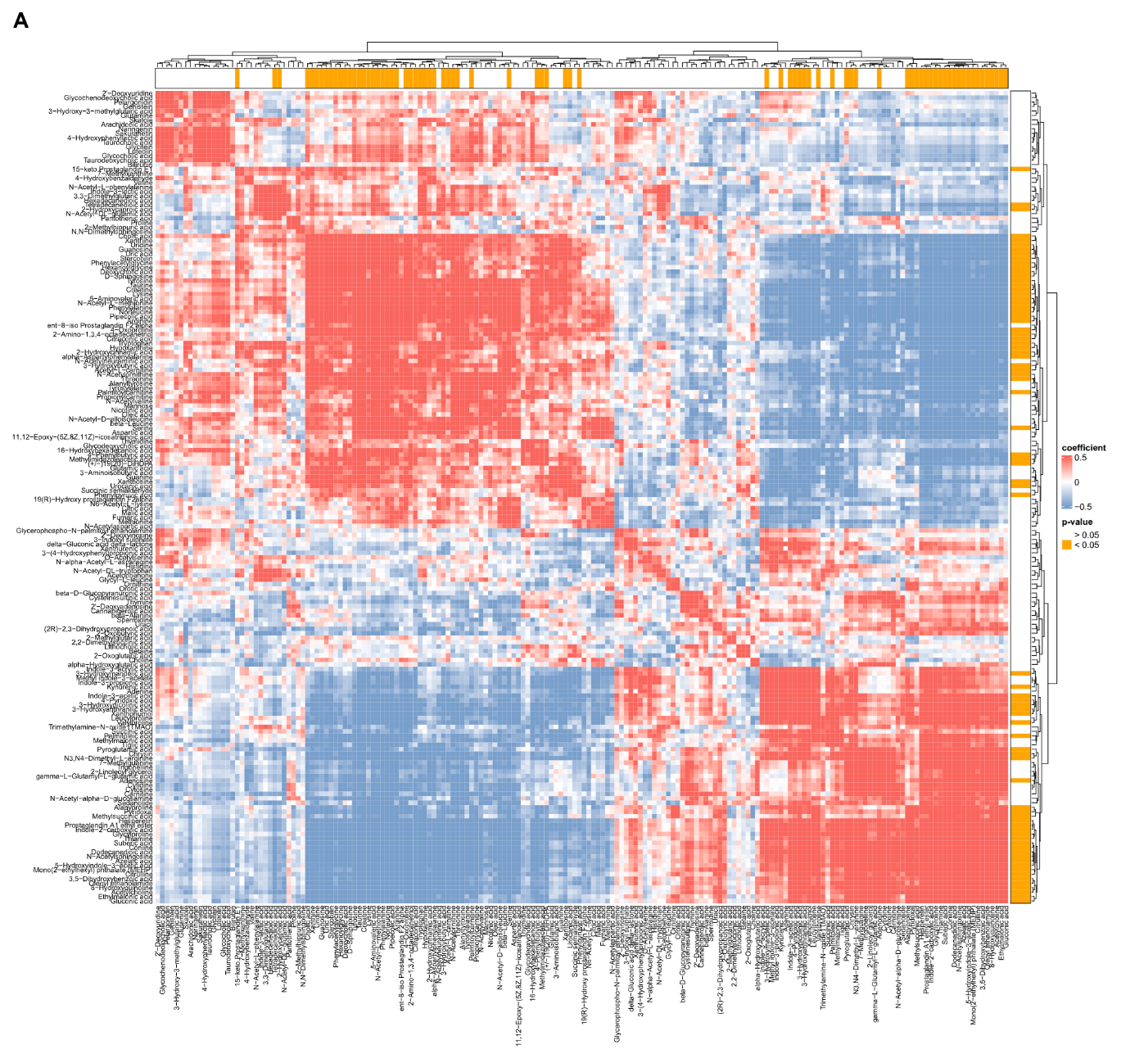


Supplementary Figure S2. Spearman correlation matrix between the metabolic features.


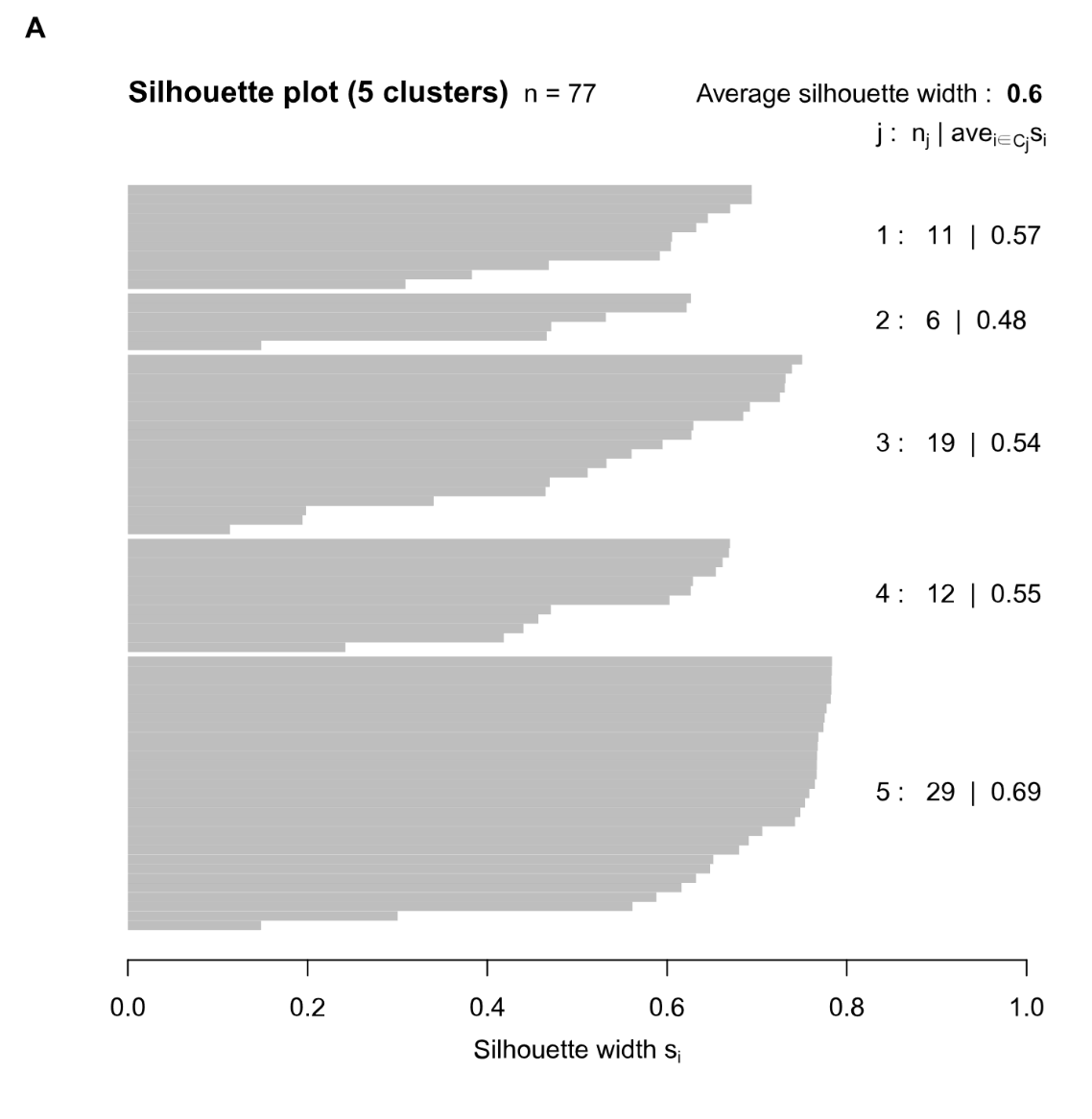


Supplementary Figure S3. Assessment of the silhouette score for the 5-clusters.


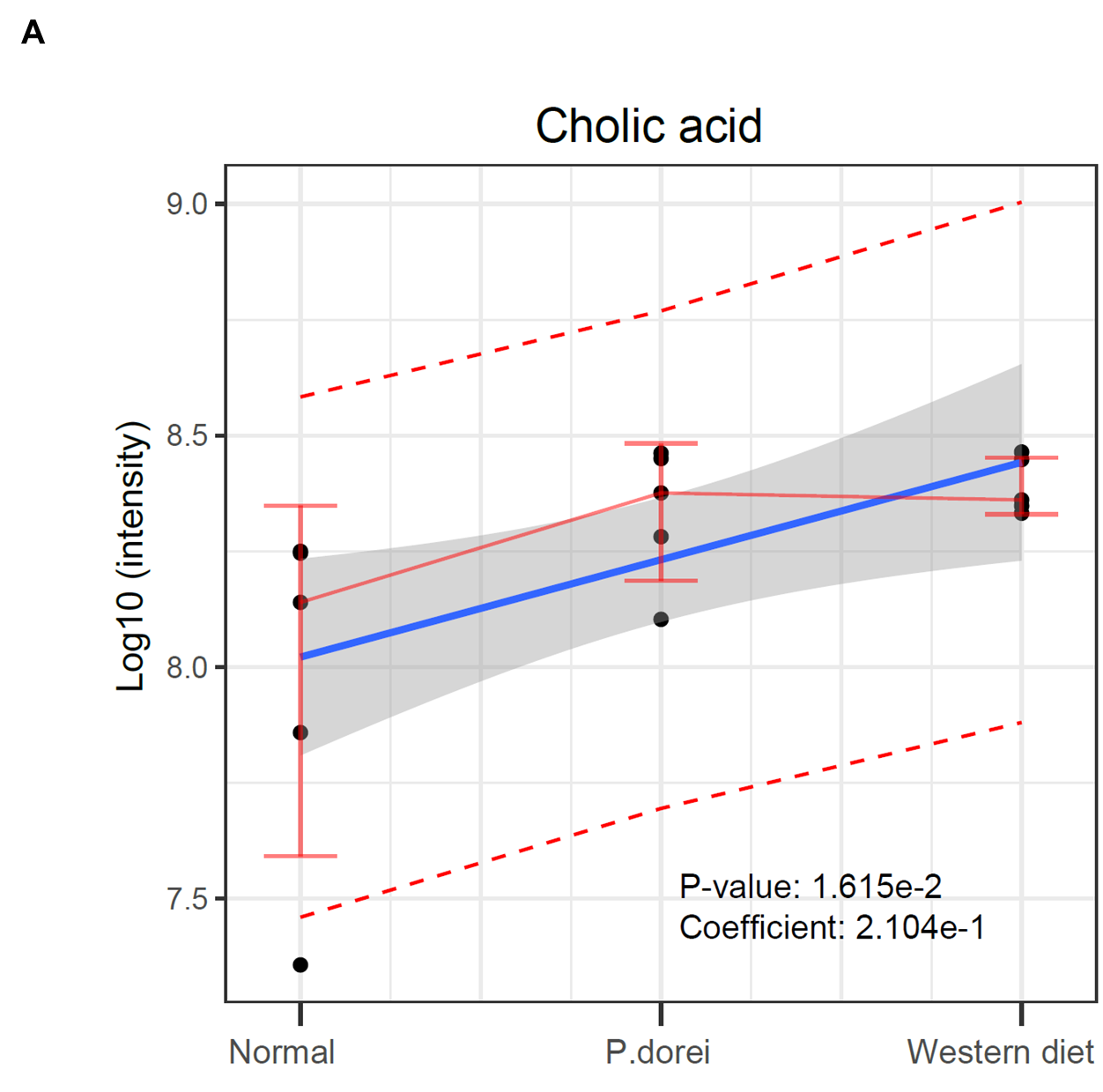


Supplementary Figure S4. Relative abundance of cholic acid in cecum samples.

**References**

1. Kleiner, D.E., Brunt, E.M., Van Natta, M., Behling, C., Contos, M.J., Cummings, O.W., Ferrell, L.D., Liu, Y.C., Torbenson, M.S., Unalp-Arida, A., et al. (2005). Design and validation of a histological scoring system for nonalcoholic fatty liver disease. Hepatology *41*, 1313-1321. 10.1002/hep.20701.

2. Caddeo, A., and Romeo, S. (2024). Precision medicine and nucleotide-based therapeutics to treat MASH. Clin Mol Hepatol. 10.3350/cmh.2024.0438.

3. Yoon, S.H., Ha, S.M., Kwon, S., Lim, J., Kim, Y., Seo, H., and Chun, J. (2017). Introducing EzBioCloud: a taxonomically united database of 16S rRNA gene sequences and whole-genome assemblies. Int J Syst Evol Microbiol *67*, 1613-1617. 10.1099/ijsem.0.001755.

4. Han, J., Lin, K., Sequeira, C., and Borchers, C.H. (2015). An isotope-labeled chemical derivatization method for the quantitation of short-chain fatty acids in human feces by liquid chromatography-tandem mass spectrometry. Anal Chim Acta *854*, 86-94. 10.1016/j.aca.2014.11.015.
